# The One Leaf (Poetry)

**Published:** 1881-04

**Authors:** 


### THE ONE LEAF.

A leaf ; a tiny, velvet leaf :
  Its colors gold and red ;
The North wind swayed the slender limb
  And then it fell off—dead.

Another year brought other leaves,
  Green and yellow and red,
But none both orange and crimson ; none
  Like this one leaf that's dead.

No mortal eye that leaf may see
  Again, save only mine :
I've pressed it, kissed it ; memory
  Will keep it fresh thro' Time.

